# The effect of internet use on depressive symptoms in older adults: based on the chain mediating role of physical exercise and self-rated health

**DOI:** 10.3389/fpubh.2024.1472445

**Published:** 2024-12-11

**Authors:** Lu Lin, Pingping He

**Affiliations:** ^1^Nursing School, Health Science Center, Hunan Normal University, Changsha, China; ^2^Nursing School, Medical College of Hengyang, University of South China, Hengyang, China

**Keywords:** internet use, the older adults, depressive symptoms, physical exercise, self-rated health, mediation analysis, potential profile analysis

## Abstract

**Background:**

Depressive symptoms in older adults have been a major public health problem. Although many studies have suggested a potential relationship between Internet use and depressive symptoms, the underlying mechanisms of this relationship among older adults remain unclear. This study aimed to explore the multiple mediating effects of physical exercise and self-rated health on the relationship between Internet use and depressive symptoms in older adults.

**Methods:**

Utilizing the 2020 data from the China Family Panel Studies (CFPS), we assessed the depressive status among the older population through the application of the Ambulatory Self-Rating Depression Scale. To validate the associations, we conducted a Pearson correlation analysis. Furthermore, we constructed a mediating effect model, which aimed to delve into the intricate pathways mediating the influence of Internet usage on depressive symptoms in the older adults. Additionally, we employed a latent class analysis to uncover the intricate heterogeneity of depression among older individuals who do not utilize the Internet, offering insights into the diverse manifestations of this condition.

**Results:**

After controlling for age, sex, marital status, chronic disease, and education level, regression results showed that the use of the Internet had a significant direct effect on depressive symptoms in the older adults (*β* = −0.684, *t* = −4.318, *p* < 0.001). Physical exercise and self-rated health significantly affected depressive symptoms in the older adults (*β* = −0.176, *t* = −7.939, *p* < 0.001; *β* = −0.937, *t* = −18.681, *p* < 0.001). Mediating results showed that the mediating effect of physical exercise between Internet use and depressive symptoms in the older adults was −0.220 (95%CI: −0.2877–−0.1598), and the mediating effect of self-rated health between Internet use and depressive symptoms in the older adults was −0.084 (95%CI: −0.1716–−0.0008), and the chain mediation effect was −0.022 (95%CI: −0.0371–−0.0076). The potential profile analysis of depressive symptoms in the older adults without using the Internet showed that they could be divided into three groups, namely, high loneliness—high depression group (7.4%), medium loneliness—moderate depression group (14.7%), and low loneliness—low depression group (77.9%).

**Conclusion:**

Internet use can directly affect the depressive symptoms of the older adults, and can also indirectly affect the depressive symptoms of the older adults through physical exercise and self-rated health. The depressive symptoms of the older adults who do not use the Internet are heterogeneous and can be divided into three categories. With the popularization of the Internet, the use of the Internet should be promoted for the older adults, and the frequency of physical exercise should be enhanced to achieve physical and mental health.

## Introduction

The 7th population census recently conducted in China shows that the proportion of the older adults aged 60 and above in the total population is as high as 18.70%, which indicates that the aging degree of China has been very serious (1). The National Health Commission subsequently issued the “14th Five-Year Plan for Healthy Aging,” which aims to cope with the current severe aging situation and enable the older adults to live a healthy and active life in old age. As China’s aging population continues to deepen, more and more people in China are facing health risks (such as loneliness, anxiety, and depression) (2). Depression, as one of the most common mental health disorders in the older adults, seriously affects the health status and quality of life of the older adults (3), and its global prevalence is as high as 7.0% (4). According to the 2019 Global Burden of Disease study, depression is one of the most disabling mental illnesses (5). The disease burden of depression not only affects the quality of life of millions of people but also presents a major health care burden (4). A nationally representative epidemiological survey of depression showed a lifetime prevalence of 7.8% for those aged 50 to 64 and 7.3% for those aged 65 and older, both higher than for younger age groups (3). Depressive symptoms are the early signs of depression. China is the country with the largest older population in the world, and sufficient attention should be paid to the depressive symptoms of the older adults (6).

With the rapid development of Internet technology in recent years, the Internet penetration rate of the older adults aged 60 and above in China has reached 43.2%, with a total of 119 million people, accounting for 11.5% of the total number of Internet users in China (7). Physical exercise refers to the process of using various sports means to exercise the body to improve health and enhance physique (8). Self-rated health is a reliable indicator for evaluating health level and a subjective evaluation based on an individual’s objective health status (9). Several studies have shown that Internet use can help reduce the level of depression in the older adults (10–12). Therefore, we hypothesized that Internet use in older adults negatively predicted depressive symptoms in older adults.

As a healthy and recreational lifestyle, physical exercise can continuously enhance the physical health level of the older adults, relieve psychological pressure, and further improve social adaptability. On the other hand, it is of great significance to enrich the emotional life of the older adults and cultivate a positive attitude toward life. Research shows that physical activity can help reduce the risk of depression, promote physical and mental health in older adults, and achieve active aging (13). In addition, there is evidence that exercise may be beneficial for people with depression, comparable to antidepressant treatment (14). Previous studies have also shown a positive correlation between Internet use and physical activity among middle-aged and older adults (15). Therefore, we hypothesized that physical exercise mediates the relationship between Internet use and depressive symptoms in older adults.

Self-rated health is a comprehensive evaluation of one’s physical condition, psychological state, and social adaptability, and is an effective factor in predicting all-cause mortality (16). Self-rated health negatively predicted depressive symptoms (17). A 3-year longitudinal study of 2,336 people found that self-rated health was a significant predictor of depression (18). Previous studies have shown that Internet use is positively correlated with self-rated health among older adults Chinese (19). Therefore, we hypothesized that self-rated health mediates the relationship between Internet use and depressive symptoms in older adults.

As we hypothesized above, physical activity and self-rated health in older adults may play a single mediating role between Internet use and depressive symptoms in older adults, respectively. However, while both physical activity and self-rated health in older adults are considered intermediaries, the relationship between them remains to be clarified. Previous research has shown that physical activity is an important predictor of depressive symptoms (20, 21). This means that the relationship between Internet use and depressive symptoms in older adults may be influenced first by physical activity and second by self-rated health. Therefore, we hypothesized that physical activity and self-rated health in older adults acted as a chain mediator between Internet use and depressive symptoms in older adults.

**Figure 1:**
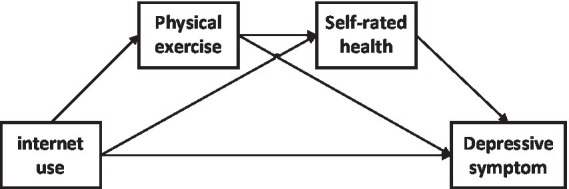
Hypothesized conceptual model of the chain mediation.

After an extensive literature review, there is a lack of research on the relationship between Internet use, physical activity, self-rated health, and depressive symptoms in older adults. However, understanding the underlying mechanisms of the effects of Internet use on depressive symptoms in older adults is critical, which could inform future studies and interventions for depressive symptoms in older adults. Therefore, to fill these knowledge gaps, this study aimed to explore the multiple mediating roles of physical exercise and self-rated health in the relationship between Internet use and depressive symptoms in the older adults, to provide the theoretical basis for the prevention and intervention of depressive symptoms in the older adults. Four hypotheses are proposed to construct the hypothesis conceptual model of this study (Figure 1).

*Hypothesis* 1: Internet use can positively predict depressive symptoms in older adults.

*Hypothesis* 2: Physical exercise mediates the relationship between Internet use and depressive symptoms in older adults.

*Hypothesis* 3: Self-rated health mediates the relationship between Internet use and depressive symptoms in older adults.

*Hypothesis* 4: Internet use and self-rated health played a chain mediating role between Internet use and nurses’ depressive symptoms.

## Methods

### Data sources and sample

**Figure 2:**
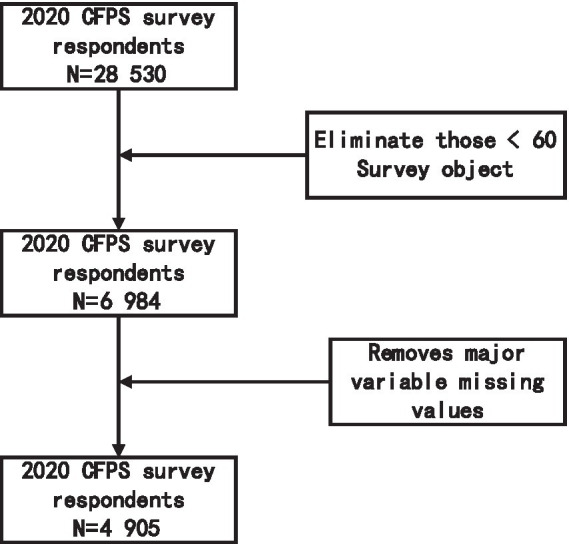
Flow chart of filtering.

The data used in this paper are from the results of the China Household Tracking Survey (CFPS) project in 2020. CFPS collects data from three dimensions: individual, family, and community, and adopts various forms of questionnaires such as long, short, pick-up, and telephone interviews, aiming to reflect the development and changes in China’s society, economy, population, health, and education. CFPS conducted initial and follow-up test investigations in Beijing, Shanghai and Guangdong in 2008 and 2009 respectively, and officially carried out visits in 2010. All baseline family members and their future biological/adopted children, as defined by the 2010 baseline survey, will be permanently tracked as genetic members of the CFPS, with surveys conducted every 2 years thereafter. In China, senior citizens refer to people aged 60 and above. Inclusion criteria: at least 60 years old; Exclusion criteria: missing variables or outliers, and 4,905 samples were obtained after screening according to the purpose of the study (Figure 2).

### Introduction and definition of variables

**Explanatory variables**: In this study, two questions in the questionnaire, whether mobile Internet access or computer Internet access, were combined to form “Whether to use the Internet.” If a “yes” answer to either of the two questions, the subject is considered to be using the Internet.

**Explained variables**: Depression symptoms were selected as the explained variables, and the depression symptoms of the older adults were measured by the mobile center Self-rating Depression Scale (CCESD-8). Participants were asked to report whether they had experienced eight symptoms for most of the past week: (1) feeling depressed, (2) feeling happy, (3) feeling lonely, (4) enjoying life, (5) feeling sad, (6) everything was an effort, (7) not being able to sleep, and (8) feeling like life could not go on. The CESD-8 scale used in this study ranges from 1 to 4 points, with a total score of 32 points, and the higher the score, the more severe the depressive symptoms. CESD-8 is widely used as a self-assessment test for the general population (22). In previous studies, the Chinese version of the scale is satisfactory in terms of structural validity and psychometric properties.

**Intermediary variables**: The mediating variables selected in this study were physical exercise and self-rated health. The question “How often have you participated in sports, fitness, and leisure activities in the past 12 months?” Definition: No participation is 0 points, from “less than once a month on average” to “more than twice a day,” respectively, from 1 to 7 points, the higher the score, the higher the frequency of physical exercise. The question “How do you consider your health?” The definition, from “unhealthy” to “very healthy” score of 1 to 5, the higher the score, the higher the perceived health level.

**Control variables**: In this paper, age, sex, marital status, chronic disease, and education level were used as control variables. Participants were defined as having a chronic disease by “Have you had a physician-diagnosed chronic disease in the past six months?”

### Data analysis

Stata MP 18.0 was used for preliminary data processing, and SPSS 27.0 was used for later data analysis. Among them, the adoption rate of counting data and measurement data are described by X ± S. Potential profiling using M plus 8.3, Aikech information criterion (AIC), Bayesian information criterion (BIC), sample corrected Bayesian information criterion (a BIC), Entropy, likelihood ratio test (LMRT), and Bootstrap-based likelihood ratio test (BLRT) were used to evaluate it. Pearson correlation analysis was used to verify the correlation between the variables, and the PROCESS 4.0 macro was used to test the mediation effect, *α* <0.05.

### Common method bias test

The Harman single-factor test was employed to scrutinize the potential common method bias among the 11 items under investigation. The outcomes indicated the presence of three distinct factors with eigenroots exceeding 1, indicating a multifactorial structure. Notably, the first common factor accounted for 31.8% of the total variance, which falls below the critical threshold of 40%. This finding provides robust evidence against the existence of substantial common method bias in the present study, assuring the validity and reliability of the data collected.

## Results

### Demographic characteristics

A total of 4,905 older people were included in this study, with an average age of 68.20 ± 5.86 years. A total of 1,103 (22.5 per cent) older persons used the Internet and 3,802 (77.5 per cent) did not use the Internet; There were 2,361 females (48.1%) and 2,544 males (51.9%); Marital status: Most the respondents (82.7%) were married and had a spouse, and 15.2% of them were widowed; Education level: The majority of them were low educated, with 19.9% having a high school degree or higher; Chronic diseases: 3442 older people (70.2%) without chronic diseases and 1,463 older people (29.8%) with chronic diseases (Table 1).

**Table 1:** Demographic characteristics (*n* = 4,905).

Variable	Options	Frequency (person)	Percentage (%)
Age (years)	60 ~ 74	4,152	84.6
	≥75	753	15.4
Gender	Male	2,544	51.9
	Female	2,361	48.1
Marital status	Never married	27	0.6
	Married (having a spouse)	4,055	82.7
	Cohabitation	15	0.3
	Divorced	61	1.2
	Widowed	747	15.2
Chronic disease	No	3,442	70.2
	Yes	1,463	29.8
Educational level	No formal education	1,585	32.3
	Primary school	1,480	30.2
	Junior high	868	17.7
	Senior high	783	16.0
	College or higher	189	3.9
Network usage	No	3,802	77.5
	Yes	1,103	22.5

### The score of each variable

The results showed that the average score of depressive symptoms was (13.59 ± 4.52). The average score of physical exercise was (1.95 ± 2.77) points; the average self-rated health score was (2.63 ± 1.25; Table 2).

**Table 2:** The score of each item.

Item	n	Mean value	Standard deviation
Depressive symptom	4,905	13.61	4.53
Physical exercise	4,905	1.96	2.78
Self-rated health	4,905	2.63	1.25

### Correlation analysis

The results showed that Internet use was positively correlated with physical exercise (*r* = 0.234) and self-rated health score (*r* = 0.064; *p* < 0.01), and negatively correlated with depressive symptoms score (*r* = −0.137; *p* < 0.01). The physical exercise score was positively correlated with self-rated health score (*r* = 0.064; *p* < 0.01), and negatively correlated with depressive symptoms score (*r* = −0.156; *p* < 0.01). Self-rated health score was negatively correlated with depressive symptom score (*r* = −0.316; *p* < 0.01; Table 3).

**Table 3:** Correlation analysis result.

Variable	Internet use	Physical exercise score	Self-rated health score	Depressive symptom score
Internet use	1.000			
Physical exercise score	0.234^**^	1.000		
Self-rated health score	0.071^**^	0.064^**^	1.000	
Depressive symptom score	−0.137^**^	−0.156^**^	−0.316^**^	1.000

**indicates significant results at the test level of 0.01.

### Analysis of the mediating effects of physical exercise and self-rated health on internet use and depressive symptoms in older adults

Model 6 in the SPSS PROCESS 4.0 macro was used to examine the mediating effect of physical exercise and self-rated health on Internet use and depressive symptoms after controlling for age, sex, marital status, chronic disease, and education level. The results showed that Internet use had a significant positive predictive effect on physical exercise and self-rated health (*β* = 1.250, *p* < 0.001; *β* = 0.089, *p* < 0.01), and negatively predicted depressive symptoms (*β* = −0.684, *p* < 0.001). Physical exercise was positively correlated with self-rated health (*β* = 0.019, *p* < 0.01), and negatively correlated with depressive symptoms (*β* = −0.176, *p* < 0.001). Self-rated health negatively predicted depressive symptoms (*β* = −0.937, p < 0.001; Table 4).

**Table 4:** Results of the mediation effect test.

Variable	Model 1: Physical exercise				Model 2: Self-rated health				Model 3: Depressive symptoms			
	SE	*β*	*t*	*p*	SE	*β*	*t*	*p*	SE	*β*	*t*	*P*
Constant	0.506	−0.541	−1.070	0.285	0.223	2.994	13.398	<0.001	0.799	22.097	27.661	<0.001
Internet use	0.100	1.250	12.443	<0.001	0.045	0.089	1.981	0.010	0.158	−0.684	−4.318	<0.001
Physical exercise					0.006	0.019	2.929	0.003	0.022	−0.176	−7.939	<0.001
Self-rated health									0.050	−0.937	−18.681	<0.001
Age	0.007	0.028	4.041	<0.001	0.003	−0.005	−1.644	0.100	0.011	−0.058	−5.386	<0.001
Sex	0.079	0.105	1.324	0.186	0.035	−0.208	5.936	<0.001	0.123	−0.660	−5.348	<0.001
Marital status	0.105	−0.093	−0.879	0.340	0.047	0.031	0.674	0.500	0.164	−1.463	−8.949	<0.001
Chronic disease	0.084	0.136	1.623	0.105	0.037	−0.829	−22.351	<0.001	0.137	0.846	6.192	<0.001
Educational level	0.089	0.762	8.556	<0.001	0.040	0.096	2.433	0.015	0.139	−0.717	−5.152	<0.001
R^2^	0.07				0.11				0.16			
F	62.29				87.67				116.25			

**Figure 3:**
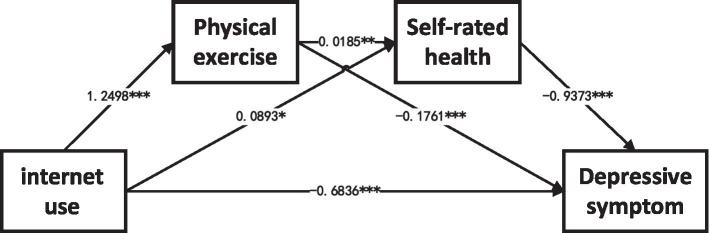
Intermediary analysis result mode. *means *p* < 0.05, **means *p* < 0.01, ***means *p* < 0.001.

The mediation analysis yielded compelling results, revealing that both the direct and indirect pathways of Internet use on depressive symptoms among the older adults were statistically significant, as evidenced by 95% confidence intervals that excluded zero. This underscores the multifaceted influence of Internet usage, which not only exerts a direct mitigating effect (with an effect size of −0.684) on depressive symptoms but also indirectly modulates them through intermediary factors such as physical exercise and self-rated health. The total effect, encompassing both direct and indirect impacts, amounted to −1.009, with the indirect effect accounting for a substantial 32.26% (−0.336) of this total, highlighting the pivotal role of these mediating factors in modulating the relationship between Internet use and depressive symptoms in the older population (Table 5; Figure 3).

**Table 5:** Effect breakdown table.

	SE	Effect size	95%CI	Effect ratio
Direct effect	0.158	−0.684	−0.9940 ~ −0.3732	67.74%
Total mediating effect	0.053	−0.336	−0.4333 ~ −0.2251	32.26%
Internet use → Physical activity → depressive symptoms	0.033	−0.220	−0.2877 ~ −0.1598	21.81%
Internet Use → Self-rated health → depressive symptoms	0.043	−0.084	−0.1716 ~ −0.0008	8.30%
Internet use → Physical activity → self-rated health → depressive symptoms	0.008	−0.022	−0.0371 ~ −0.0076	2.15%
Total effect	0.138	−0.1009	−1.3274 ~ −0.6907	100%

### Potential profiling of depressive symptoms in older adults without using the internet

As the number of profiles increased, the AIC, BIC, and aBIC values consistently declined, indicating improved model fit. Notably, the Entropy values peaked at 3 profiles, accompanied by a statistical significance of *p* < 0.001, providing robust evidence for selecting this number of profiles. Consequently, a comprehensive analysis led to the selection of 3 profiles.

**Figure 4:**
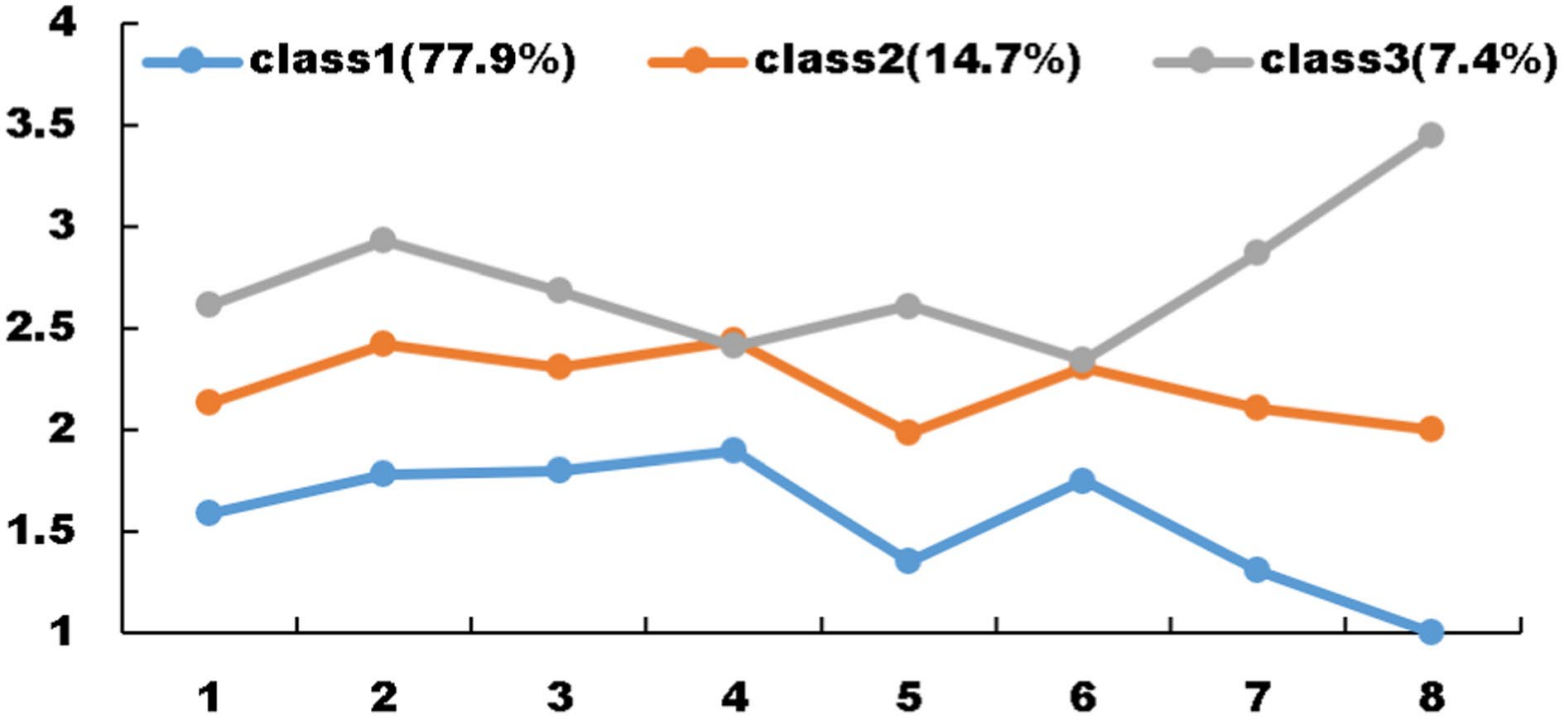
Distribution of each type of model 4.

Based on the item scores within each group, they were aptly named: the high loneliness—high depression group, comprising 7.4% of the participants; the medium loneliness—moderate depression group, accounting for 14.7%; and the low loneliness—low depression group, which constituted the largest proportion at 77.9%. This categorization offers a nuanced understanding of the varying degrees of loneliness and depression experienced by the older adults (Table 6; Figure 4).

**Table 6:** Fitting indexes of 1–5 profile solutions in potential profile analysis.

Medel	AIC	BIC	aBIC	Entropy	P(BLRT)	Class probability
1	80405.243	80505.136	80454.295			1.00000
2	74187.779	74343.861	74264.423	0.905	<0.001	0.80654/0.19346
3	68891.926	69104.198	68996.162	1	<0.001	0.77933/0.14703/ 0.07365
4	66837.095	67105.557	66968.923	0.925	<0.001	0.15413/0.14703/0.62520/0.07365
5	65961.574	66286.225	66120.993	0.910	<0.001	0.12707/0.09516/0.55710/0.14703/0.07365

## Discussion

This study was designed to explore the multiple mediating effects of physical exercise and self-rated health on the relationship between Internet use and depressive symptoms in older adults. The results of this study confirm a direct relationship between Internet use and depressive symptoms in older adults. At the same time, this study confirmed that Internet use affected depressive symptoms in older adults in two ways: (a) Internet use → physical exercise → depressive symptoms, and (b) Internet use → physical exercise → self-rated health → depressive symptoms.

For hypothesis 1, this study confirms that Internet use has a direct positive effect on depressive symptoms in older adults, compared with the older adults who use the Internet, the older adults who do not use the Internet have more serious depression symptoms, which is consistent with the research results of Ju et al. (23). On the one hand, the Internet can help the older adults maintain parent–child relationships and family relationships, maintain interaction with friends (24), broaden the scope of interpersonal communication and social participation, and strengthen social contact with others (25). On the other hand, the Internet provides health-related information and entertainment resources for the older adults (26) to improve their health.

For hypothesis 2, this study confirms that physical exercise mediates the relationship between Internet use and depressive symptoms in older adults. First, the use of the Internet may help the older adults obtain information on how to do moderate physical activity and promote healthy and effective physical exercise (27). A cross-sectional study in Japan showed that Internet use in older adults was associated with voluntary exercise (28). In addition, Internet use is affected by economic status (29), and some studies have found that older adult men with poor economic status may be less likely to take physical exercise (30), which also explains the predictive effect of Internet use on physical exercise to a certain extent.

For hypothesis 3, we found an association between self-rated health-mediated Internet use and depressive symptoms in older adults. Multiple studies have shown that Internet use has a positive impact on the self-rated health of the older adults (31, 32). On the one hand, the older adults can obtain health care related information through the Internet or consult health care personnel on the Internet, which is conducive to the health promotion of the older adults; On the other hand, the older adults can obtain a large number of entertainment resources by using network equipment, such as small videos, games, news headlines, etc., which adds a touch of color to the older adults life to a certain extent.

For hypothesis 4, this study confirmed the sequential mediating effect of physical exercise and self-rated health on the relationship between Internet use and depressive symptoms in older adults. Sequential mediating effects suggest that older adults who do not use the Internet may be less likely to engage in physical activity, which may lead to lower self-rated health and ultimately worse depressive symptoms. Physical exercise can negatively predict depressive symptoms, which is consistent with previous research conclusions (33–35). It may be related to the fact that physical exercise improves mood through changes in endorphin and monoamine levels or decreased levels of the stress hormone cortisol (36). Self-rated health can negatively predict depression symptoms in the older adults, which is consistent with the research results of Zhang et al. (37). Self-rated health is the older adult subjective evaluation of their health status, which reflects their current objective health status to a large extent (38). Older adults without depressive symptoms may be more likely to have a positive attitude and evaluation of their health than older adults with more severe depressive symptoms. Therefore, there is a need to pay attention to Internet use and its series of knock-on effects, namely reduced levels of physical activity and self-rated health, to prevent and intervene in the older adults with depressive symptoms promptly.

### Potential types of depressive symptoms in older adults without internet use

This study unveiled a noteworthy heterogeneity in depressive symptoms among older individuals who abstain from Internet usage. The participants were categorized into three distinct groups: a high loneliness-high depression cohort accounting for 7.4%, a medium loneliness-moderate depression group comprising 14.7%, and a low loneliness-low depression segment making up 77.9% of the sample. Notably, individuals belonging to the high loneliness-high depression group exhibited elevated scores across all assessed dimensions, with ‘feeling lonely’ scoring the highest among all three groups, signifying a more severe manifestation of depressive symptoms and a profound sense of isolation. Conversely, those in the medium loneliness-moderate depression group demonstrated scores at a moderate level, indicating a corresponding intermediate degree of depressive symptoms. Lastly, the low loneliness-low depression group consistently recorded lower scores, with ‘feeling lonely’ also being the lowest among the three, underscoring a lesser burden of depressive symptoms within this group. The prominence of ‘feeling lonely’ as a common denominator across these groups underscores the critical need for medical professionals to prioritize the mental health and living conditions of the older adults. Enhanced efforts should involve dispatching more volunteers to provide companionship, organizing diverse activities to mitigate loneliness, and promoting both physical and mental wellness. By addressing these issues holistically, we can strive toward the achievement of healthy aging for all.

In essence, harnessing the Internet’s potential has demonstrated a capacity to alleviate depressive symptoms among older adults, primarily by fostering physical exercise and enhancing self-assessed health. However, the current landscape reveals substantial untapped potential in augmenting the older population’s Internet penetration rate. Notably, a significant portion of seniors are either unfamiliar with or lack access to the Internet, impeding their full integration into contemporary society. Thus, it is imperative to intensify efforts in educating and training seniors on Internet usage, devising older adults-friendly digital tools, establishing dedicated online platforms tailored to their needs, and furnishing them with pertinent health resources and recreational amenities. These initiatives aim to bridge the digital divide and ensure that no one is left behind. Intriguingly, the intermediary role of physical exercise in mediating the relationship between Internet usage and depressive symptom relief is notably more pronounced than that of self-rated health. This underscores the importance for healthcare professionals to actively encourage and empower seniors to embrace the Internet, enhancing their digital literacy, and thereby facilitating a more vigorous promotion of physical activity. This comprehensive approach aims to optimize both the physical and mental well-being of our older adults, fostering a life of happiness and contentment. Furthermore, heterogeneity studies reveal nuanced findings: the mediating effect is non-existent among unmarried older adults while varying significantly across age, gender, chronic illness status, and educational attainment. These insights underscore the necessity for healthcare providers to adopt a tailored, condition-specific approach when devising intervention strategies, thereby maximizing their effectiveness and impact.

### Implications for future practice

In the context of accelerated aging, it is of great significance to pay attention to the mental health of the older adults to realize active aging. Our findings provide practical implications for prevention and intervention of mental health in older adults. Firstly, the government should pay attention to the Internet use of the older adults to reduce the risk of depression in the older adults. For example, we can organize educational activities related to the use of the Internet to teach the older adults to use the Internet correctly, so that the older adults can better integrate into the Internet era. Secondly, for the older adults who do not use the Internet, medical staff should pay more attention to their mental health. For example, regular physical exercise and emotional communication can be organized through volunteer activities to prevent or reduce depressive symptoms in the older adults. Finally, health care providers can regularly assess older adults’ Internet use, physical activity, self-rated health, and depressive symptoms, and develop targeted prevention and interventions for depressive symptoms in older adults.

### Limitations and future research directions

This study also has some shortcomings. For the use of the Internet, only the classification of “whether to use,” and no further research on the frequency of use, time of use, purpose of use and so on. Secondly, this study is a cross-sectional study and lacks the verification of variation and causality between variables. In addition, most of the subjects included in this study did not suffer from chronic diseases, and the risk of chronic diseases is related to the use of the Internet, which may cause a certain bias in our research results. It is recommended that years of data be included in future studies and that longitudinal and causal studies be conducted. The association of chronic diseases with Internet use and depressive symptoms can also be explored in depth.

## Conclusion

This study shows that Internet use has a direct positive effect on depressive symptoms in older adults. At the same time, Internet use may influence depressive symptoms in the older adults through the mediating effect of physical exercise and the chain mediating effect between physical exercise and self-rated health. In addition, this study conducted a potential profile analysis of depressive symptoms in older adults who did not use the Internet and found heterogeneity, which could be divided into three groups: high loneliness—high depression, moderate loneliness—depression, and low loneliness—low depression. This is the first potential profiling of depressive symptoms in older adults who do not use the Internet. These findings may help healthcare professionals better understand the underlying mechanisms between Internet use and depressive symptoms in older adults, and develop targeted prevention and interventions for depressive symptoms in older adults in the future.

## Data Availability

Publicly available datasets were analyzed in this study. This data can be found at: http://www.isss.pku.edu.cn/cfps/.
